# An Elaboration on Sample Size Planning for Performing a One-Sample Sensitivity and Specificity Analysis by Basing on Calculations on a Specified 95% Confidence Interval Width

**DOI:** 10.3390/diagnostics13081390

**Published:** 2023-04-11

**Authors:** Mohamad Adam Bujang

**Affiliations:** Clinical Research Centre, Sarawak General Hospital, Ministry of Health Malaysia, Kuching 93586, Malaysia; adam@crc.gov.my

**Keywords:** confidence interval, sample size, sensitivity, specificity

## Abstract

Sample size calculation based on a specified width of 95% confidence interval will offer researchers the freedom to set the level of accuracy of the statistics that they aim to achieve for a particular study. This paper provides a description of the general conceptual context for performing sensitivity and specificity analysis. Subsequently, sample size tables for sensitivity and specificity analysis based on a specified 95% confidence interval width is then provided. Such recommendations for sample size planning are provided based on two different scenarios: one for a diagnostic purpose and another for a screening purpose. Further discussion on all the other relevant considerations for the determination of a minimum sample size requirement and on how to draft the sample size statement for performing sensitivity and specificity analysis are also provided.

## 1. Introduction

Diagnostic research is one of the most popular types of research in the medical field. It is a study that aims to quantify the accuracy of a test’s added contribution beyond the test results readily available to the physician or researcher in determining the presence or absence of a particular disease or to predict the two distinct categories of patients such as poor health or good health [1,2,3,4,5,6,7]. This type of study is very important for efficiently identifying and offering the appropriate medical management to the right patient [8,9,10].

**Figure 1:**
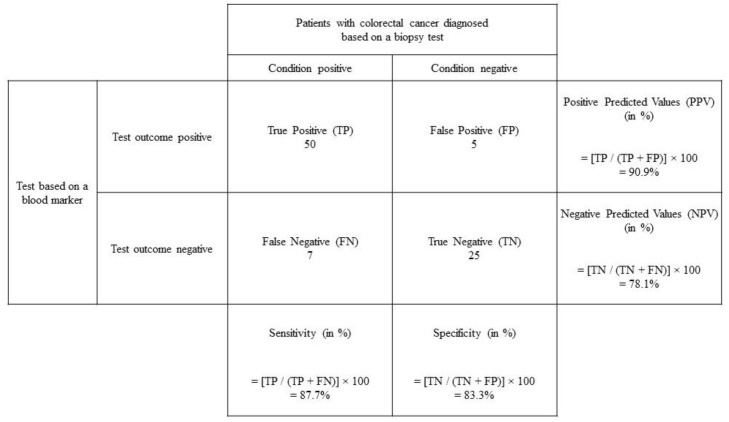
Four possible scenarios in diagnostic research.

Research design is one of the important factors that will define the success of diagnostic research, and one of the necessary considerations for any research design is to conduct proper sample size planning. Before calculating the required sample size, a researcher will need to first understand the overall concept, the underpinning assumptions, and all the measurable parameters for a diagnostic test. Figure 1 illustrates a common scenario for diagnostic research. In this example, the researcher aims to determine the accuracy of a particular screening test to determine the serum level of a particular biochemical marker for detecting colorectal cancer in a patient. The outcome of a diagnostic test must be objectively evaluated against a definitive measurement provided by a gold standard test such as in this case from a biopsy test.

The True Positive (TP) cases are referring to those cases that actually have a positive diagnosis from among a group of positive cases detected by the test, whereas the True Negative (TN) cases are referring to those cases that actually do not have a positive diagnosis from among a group of negative cases detected by the test. This means that the sensitivity of a diagnostic or screening test is an assessment of how well it is able to detect the True Positive (TP) cases (e.g., patients with colorectal cancer) as compared to that of the gold standard technique (i.e., performing a biopsy from the organ itself); whereas, the specificity of a diagnostic or screening test is an assessment of how well it is able to detect the True Negative (TN) cases (e.g., patients without colorectal cancer) as compared to that of the gold standard technique (i.e., performing a biopsy from the organ itself). In other words, a diagnostic or screening test with a perfect score in having both the sensitivity and specificity values of 100%, respectively, can only be achieved when the False Positive (FP) and False Negative (FN) are both zero.

Based on the above formula provided, the sensitivity and specificity of the test are calculated to be 87.8% and 83.3%, respectively. The Positive Predicted Value (PPV) is the proportion of people with a positive test result who actually have the disease and Negative Predicted Value (NPV) is the proportion of those with a negative result who do not have the disease. In this example, the values of PPV and NPV are then calculated to be 90.9% and 78.1%, respectively. Overall, the test has good sensitivity and specificity. Ideally, most researchers will always aim to achieve a perfect accuracy, which is a performance as good as the gold standard. However, this can rarely be achieved since a particular screening test that has been invented or developed will usually be far cheaper, offer a faster method of detection, and be more convenient and user-friendly in its procedures. Thus, most researchers will usually afford some allowances for its accuracy that are attributable to chance or random error [8,9,10].

Normally, there are three possible conclusions that can be drawn from diagnostic research. First, the test is both sensitive and specific and thus suitable for use as a diagnostic test or marker [1,2,3,4,5]. Second, the test can only be suitable for use as a screening tool since the test or marker is high in either its sensitivity or specificity (but not both) but is low in the other measures [11,12,13,14,15,16,17]. Lastly, the test is neither sensitive nor specific and perhaps this is the worst-case scenario in diagnostic research, which renders it not being suitable for use in either the diagnosis or screening of a disease [18,19,20]. The ideal result is to obtain an excellent measure for both its sensitivity and specificity or at least in one of its two evaluated measures (i.e., sensitivity or specificity) so that the test can still be deemed acceptable for use as a screening tool at a bare minimum.

This paper adopts this position further by proposing that a careful evaluation of the actual purpose of diagnostic research (for either diagnosis or screening of a disease) is necessary because both purposes are not the same and each will require a different approach in its sample size planning. Many previous studies have provided the detailed estimation or calculation of sample size requirement for the purpose of sample size planning when conducting diagnostic tests as presented in Table 1. Although there are already numerous published papers related to sample size planning for performing sensitivity and specificity tests, it is still necessary to provide further detailed step-by-step guidance of how to apply the relevant knowledge to ensure the researchers do not inadvertently omit accounting for any other pertinent considerations during the sample size planning for conducting diagnostic research. Furthermore, the sample size determination must also be guided by the specific study objective that is the aim of a particular diagnostic test (and also its expected level of accuracy).

Therefore, this study shall further extend the aim for determining the necessary sample size requirement in these situations by discussing the detailed step-by-step procedures of sample size planning for diagnostic research through the incorporation of a specified width of both sensitivity and specificity values that are based on a 95% confidence interval. The advantage of using the width as a proxy measure for its effect size is to enable the researcher to impose a pre-specified limit for its sensitivity and specificity values based on a 95% confidence interval that the researcher initially aims to achieve. By doing so, a list of sample size tables will be compiled to guide the researcher by facilitating them to set the sample size requirement by quickly conducting the necessary sample size planning without the need to understand the complexity of the computations involved.

## 2. Methods

The sample size calculations were determined by basing on two-sided confidence intervals for conducting a one-sample sensitivity and specificity analysis [35]. The formula for calculating the binomial confidence intervals was derived from an ‘exact’ method called the Clopper–Pearson interval in which these intervals are being calculated by directly basing them on the cumulative probabilities of the actual binomial distribution [38]. For all these calculations, the alpha is set at 0.05, confidence interval width is set at 0.1 or 0.2, and the prevalence is set at 0.05, 0.1, 0.2, 0.3, 0.4, 0.5, 0.6, 0.7, 0.8, or 0.9.

For a study design that aims for diagnostic purposes, the values of both sensitivity and specificity are set at 0.7, 0.8, 0.9, and 0.95, respectively. A diagnostic test or marker should ideally have excellent levels of both sensitivity and specificity. In this paper, the minimum value of sensitivity and specificity is set at 0.70. For ease of interpretation, the values of both sensitivity and specificity of 0.95 can be regarded as having excellent accuracy, 0.90 as having nearly excellent accuracy, 0.80 as good accuracy, and 0.70 as fairly good accuracy.

For a study design that aims for screening purposes that place a particular emphasis on sensitivity, the pre-specified sensitivity values are set at 0.95, 0.90, 0.80, and 0.70 while the same for specificity is set at 0.5. Meanwhile, for a screening with a particular emphasis on specificity, the pre-specified specificity values are set at 0.95, 0.90, 0.80, and 0.70 while the same for sensitivity is set at 0.5. To develop a screening strategy, it might be necessary for the researchers to have to sacrifice either the sensitivity or specificity. In the case where a researcher has initially planned to ensure that a study must have a high level of sensitivity, the minimum setting for its sensitivity will be at least 0.7 while the minimum setting for its specificity is 0.5. All the calculations were performed by using Power and Sample Size Software (PASS) (PASS 2020 Power Analysis and Sample Size Software (2020). NCSS, LLC. Kaysville, UT, USA, ncss.com/software/pass).

## 3. Results

There are three main factors that can potentially contribute to the requirement of a larger sample size. Firstly, the determination of smaller values for sensitivity and specificity will usually command a larger sample size requirement. Secondly, the prevalence of a disease or outcome of interest will dictate the sample size requirement in that a lower prevalence will necessitate a larger sample size requirement for a determination of its sensitivity, whereas a higher prevalence will demand a larger sample size for a determination of its specificity. Thirdly, a narrower desired half-width of the confidence interval (which is equivalent to a smaller marginal error) will also command a bigger sample size requirement. Hence, an ‘ideal’ sample size will not be available for which it can be universally applied because the determination of an ‘ideal’ sample size shall ultimately depend on the conditions and prerequisites for the setting up of the target effect size (Table 2, Table 3 and Table 4).

For a diagnostic purpose, the researcher will usually aim to have an excellent level of both sensitivity and specificity. Therefore, these sample size calculations are now presented in Table 2, which provides a pair of same values for both sensitivity and specificity. Based on the initial setting of the requirements for its target sensitivity and specificity, the minimum sample size requirement can range from 58 to 26,580 subjects. The ideal goal for a researcher is to achieve an excellent level of accuracy (i.e., to aim for both sensitivity and specificity of at least 0.95) and, hence, only a smaller sample size will usually be required for recruitment. However, by considering the highly probable risk of not being able to reach an excellent level of both sensitivity and specificity, a researcher will be encouraged to recruit more subjects to ensure that he/she is able to confidently conclude that the reported level of accuracy of a particular diagnostic condition is at least satisfactory (i.e., with a degree of sensitivity and specificity of at least 0.80).

For a screening purpose, the researcher will usually aim to have achieved an excellent level of either sensitivity or specificity, but not both. To facilitate the setting up of all conditions and prerequisites for conducting the proper sample size planning of a screening condition, the tabulation of all these sample size calculations is now presented in Table 3 and Table 4. Most studies that emphasize a screening purpose will aim for a higher degree of sensitivity and, thus, they may need to sacrifice their specificity levels. A list of various pre-specified values for their sensitivity are now provided with its minimum value of 0.70% along with a fixed pre-specified specificity value of 50.0% (Table 3). The ideal goal for a researcher is to set to achieve an excellent degree of sensitivity such as 0.95. Based on the tabulated values displayed by Table 3, this means that if a researcher decides to set the desired width of a 95% confidence interval to be 0.20, then its minimum sample size requirement shall range between 174 and 1041 depending on the prevalence of the disease or outcome of interest.

## 4. Discussion

Scholars have developed numerous techniques to estimate or calculate a minimum sample size requirement for diagnostic research. There is no single technique that is superior to others because it totally depends on the study’s purpose and the researchers’ expectations. The sample size issues regarding diagnostic research were first discussed by Linnet in 1987 and, after that, the discussion regarding sample size is still continuing to be discussed with dozens of articles being published to discuss this matter even further [21,22,23,24,25,26,27,28,29,30,31,32,33,34,35,36,37]. The summary of these findings was described in Table 1.

Previous studies had found some spurious findings that were being derived from research pertaining to sample size planning for diagnostic research, such as the requirement of a very small minimum sample size [39,40]. In order to avoid the possibility of misconstruing the statistical rigor inherently present in this type of analysis, this paper adopts a different approach by offering sample size tables for performing sensitivity and specificity analysis that are based on the use of desired width of 95%CI as a measure for clinical or scientific significance, and are hence emphasizing the importance of this desired interval width as the level of confidence in all types of diagnostic research [35].

By imposing a tighter limit on the desired width of 95%CI (i.e., 0.1 or 0.2), the researcher will be more confident in ensuring that the accuracy of the study can realistically be scientifically justifiable. It is a well-known fact that a statistically significant result (i.e., *p* < 0.05) can be erroneously caused by an extremely large sample size [41]. Thus, some scholars may argue over the utility of the *p*-value, but it is nevertheless still applicable and acceptable until now [42,43,44,45]. Therefore, by imposing an additional condition such as the placement of a relevant fixed limit for the desired width of 95%CI; it is less likely for the researcher to be misguided and hence they will be better able to ensure that an acceptable level of accuracy can be realistically achieved.

### 4.1. How Should the Sample Size Tables Be Used?

The sample size tables are provided in this paper to facilitate a researcher for the purpose of sample size planning of all studies related to sensitivity and specificity analysis. Firstly, a researcher needs to determine the prevalence of a disease or the outcome of interest (such as ‘poor outcome’ or ‘good outcome’). The prevalence of a disease per se can vary widely depending on which type of study population a researcher aims to study. In other words, the researcher shall have to decide the specific type of study population for which a diagnostic or screening condition is intended. For example, the prevalence of colon cancer among a ‘high-risk’ population is obviously very much higher than that among a healthy population. If the researcher aims to implement the diagnostic or screening test or marker among the ‘high-risk’ population for colon cancer, then an estimate of the prevalence of colon cancer in the study population should be calculated from the ‘high-risk’ population for colon cancer (i.e., patients from a hospital setting such as the surgical specialist clinic).

Secondly, the researcher needs to decide whether the test or marker is intended to be an alternative for a diagnostic tool/marker or will solely be used for screening purposes. As a researcher or research scientist, they should be able to decide the desired aim of a particular test or marker since they are also the subject matter in the specialized field and should therefore know the true capabilities and expectations of a diagnostic test or marker. Hence, Table 2 should be referred to if a researcher intends to develop a diagnostic test or marker, whereas Table 3 and Table 4 should be referred to if they intend to develop a screening test or marker.

Thirdly, the researcher also needs to decide beforehand the target values of both the sensitivity and specificity of a test or marker. If they intend to develop a diagnostic test or marker, then there will be four different possible sets of sensitivity and specificity values. For the sake of simplicity, this paper thereby recommends that both the sensitivity and specificity are being measured by a score of 0.95, 0.90, 0.80, and 0.70 to be regarded as an excellent, nearly excellent, good, and fairly good diagnostic test/marker, respectively. Finally, the researcher will also have to decide beforehand the desired interval width of 95%CI (i.e., either 0.1 or 0.2). The determination of the desired interval width is likely to be driven by the actual intended purpose of the study, the availability of resources, and the capability and experience level of the researcher under the various experimental conditions.

Say, for example, the prevalence of a disease is set at 40.0%, the target desired interval width for 95%CI is set at 0.1 and the desired degrees of sensitivity and specificity are set at 0.90, respectively. Based on the abovementioned conditions, the minimum required sample size to perform an analysis for the determination of sensitivity is 395 and that for the determination of specificity is 264. In this case, the sample size of 395 shall be preferably chosen since it yields a much larger sample than the other. In another scenario, say, for example, the prevalence of a disease is set at 70.0%, the target desired interval width for 95%CI is set at 0.1 and the desired degrees of sensitivity and specificity are both set at 0.90, respectively. Based on such conditions, the minimum required sample size for assessing the degree of sensitivity is 135 and that for assessing the degree of specificity is 1304. Again, in this case, the sample size of 1304 shall be chosen preferably for the same reason mentioned above.

### 4.2. Issues That Can Arise from Very Large Sample Sizes Involving Very Low Level of Prevalence

It is evident that some of the calculations in the tables have yielded extremely large sample size requirements. For example, Table 2 has shown that a minimum of 26,580 subjects will be needed to claim for the degree of both sensitivity and specificity of 70.0%, which are based on the desired interval width of 95%CI of 0.05 in a study population with a 5.0% prevalence rate of disease. There are two main pertinent issues that await our due consideration here. Firstly, it is necessary to carefully consider whether the purported values of sensitivity and specificity of 70.0% will satisfy both the researchers and stakeholders (who are the end-users of the test or marker) and, secondly, it is also necessary to determine whether or not the researchers can realistically cope with the work involved in the recruitment for a very large number of subjects.

This means to say that the recruitment of a large number of subjects will only be regarded worthwhile if the study can realistically be proven to be very highly sensitive and specific, such as having an exceptionally high degree of both sensitivity and specificity at 95.0%. In other words, it is only recommended to recruit an unusually large number of subjects if there are sufficient grounds for us to believe that a diagnostic test or marker demonstrates a very high level of high accuracy. Such grounds can often be retrieved from the literature or they can be based on cumulative scientific evidence for an evaluation of accuracy of the test marker.

Thus, the most important consideration here is that the core emphasis for diagnostic research shall be to develop a sensitive and specific marker by garnering sufficient cumulative evidence of its sensitivity and/or specificity and not just to merely study a particular diagnostic test/marker for its sensitivity or specificity without having accruing sufficient evidence of its sensitivity and specificity.

In other words, it is not recommended to conduct a study with very large number of subjects merely to prove that a diagnostic test/marker has a degree of both sensitivity and specificity of 70.0%. However, these calculations are being presented in this paper merely to illustrate the point that the recruitment of such a high number of subjects can be justifiable if and only if the accruing evidence has already demonstrated sufficient grounds that a particular diagnostic/screening test or marker has garnered cumulative scientific evidence of a high level of sensitivity and/or specificity, which provides a valid rationale for the study [46,47,48,49,50]. Otherwise, it is not recommended to do so.

### 4.3. Determination of Sample Size Requirements for Diagnostic Purposes

One previous study provided a list of recommended criteria for creating a sample size statement that should ideally include five elements. These elements shall consist of Step 1: to understand the objective of the study, Step 2: to select the appropriate statistical analysis, Step 3: to calculate or estimate the sample size, Step 4: to provide additional allowances during the subject recruitment procedure to cater for a certain proportion of non-response, and Step 5: to write a standard sample size statement [51]. For the purpose of writing a standard sample size statement, a common scenario has been created as follows: the researchers aim to prove that a particular new marker extracted from a patient’s blood is suitable for use as a diagnostic marker to determine whether the patient has colon cancer.

Thus, the sample size statement is written as follows: “This study aims to determine whether marker X is highly accurate to detect all patients with colon cancer. The basis of its sample size calculation is derived from both sensitivity and specificity analyses. In a population at risk of colon cancer (i.e., patients who have already exhibited and reported to have usual symptoms of colon cancer), the prevalence of colon cancer is 10.0%. For a reliable diagnostic marker, the researcher will typically aim the new marker to have a degree of both sensitivity and specificity of at least 95.0%. The sample size calculation is based on the desired width of the 95% confidence interval for both its sensitivity and specificity to be set at 1.0. Based on the abovementioned conditions, the minimum sample size requirement to perform a study for determining its sensitivity is 940 patients and that for determining its specificity is 105 patients. Therefore, the minimum sample size of 940 patients shall be deemed necessary since it yields a larger sample between the two. In order to provide additional allowances for incorporating a possible non-response rate of 20.0%, the minimum required sample size is then further inflated to 1175 patients.”

### 4.4. Determination of Sample Size Requirements for Screening Purposes

Yet, another similar scenario can be applied for the following example whereby the researcher is now aiming to prove that a particular new marker extracted from a patient’s blood is suitable for use as a screening marker (i.e. equal or more than 70.0% for its sensitivity) for colon cancer with the fixed degrees of 50.0% for its specificity or vice versa. Thus, the sample size statement is written as follows: “This study aims to determine whether marker Y is highly sensitive to screen a patient for the purpose of detecting colon cancer. The basis of its sample size calculation is derived from both sensitivity and specificity analyses. In a population at risk of colon cancer (i.e., patients who have already exhibited and reported to have usual symptoms of colon cancer), the prevalence of colon cancer is 10.0%. To obtain a reliable screening marker, the researcher will typically aim for the new marker to have a degree of sensitivity of at least 95.0% and that of specificity of at least 50.0%. The sample size calculation is based on the desired width of a 95% confidence interval for both its sensitivity and specificity to be set at 2.0. Based on the abovementioned conditions, the minimum sample size requirement to perform a study for determining its sensitivity is 290 patients and that for determining its specificity is 116 patients. Therefore, the minimum sample size of 290 patients shall be deemed necessary since it yields a larger sample between the two. In order to provide additional allowances for incorporating a possible non-response rate of 20.0%, the minimum required sample size is then further inflated to 363 patients”.

### 4.5. Conclusions

Researchers often need a quick and simple ‘rule-of-thumb’ or method to estimate or calculate the minimum sample size requirement. This paper provides background information on a diagnostic study, a list of sample size tables for determining the minimum sample sizes required for performing both the sensitivity and specificity analysis together with a clear and concise guideline on how to use the sample size tables for performing such analysis under a wide variety of differing conditions, and, lastly, it wraps up the whole discussion by offering an illustrative example of how a standard sample size statement should be written.

Indeed, this paper provides a recommendation that the researcher shall now have to set a tighter desired width for the 95% confidence interval (i.e., 0.1 or 0.2) for better sample size planning. All in all, this paper will assist the researcher to conduct a proper sample size planning related to diagnostic research and, hence, it facilitates the researcher to reach a simple and quick decision on sample size planning without resorting to the use of many highly complicated statistical techniques for their computations, as well as to a formal in-depth acquisition of the knowledge and technicality of the subject matter.

## Figures and Tables

**Table 1:** Summary of existing published literature related to conducting a proper sample size planning for performing sensitivity and specificity analysis.

No.	Authors	Year	Content
1	Linnet [21]	1987	Comparison of quantitative diagnostic tests
2	Simel et al. [22]	1991	Emphasizes sample size based on likelihood ratios
3	Burderer [23]	1996	Incorporating the prevalence of disease into the sample size calculation
4	Carpenter and Gardner [24]	1996	Determine herd-level predictive values and sensitivity based on test sensitivity, specificity, and sample size
5	Obuchowski and McClish [25]	1998	Sample size calculation involving binomial ROC curve indices
6	Lui and Cumberland [26]	2001	Provide sample size determination for equivalence test using rate ratio of sensitivity and specificity in paired sample data
7	Dendukuri et al. [27]	2004	Sample size calculation using Bayesian technique
8	Li and Fine [28]	2004	Develop a sample size and power calculations based on the unconditional power properties of the test statistics
9	Flahault et al. [29]	2005	Calculation emphasizes the study design such as requirements for the selection of cases and controls
10	Carley et al. [30]	2005	Provide nomograms for sample size planning
11	Moskowitz and Pepe [31]	2006	Comparative inference about predictive values of diagnostic tests for paired study design
12	Steinberg et al. [32]	2008	Sample size calculation emphasizes the predictive predicted value and negative predicted value
13	Fosgate [33]	2009	Sample size emphasizes surveillance and diagnostic investigations
14	Malhotra and Indrayan [34]	2010	Provide nomograms for sample size planning
15	Tilaki [35]	2014	Sample size calculation based on prevalence and marginal errors
16	Bujang and Adnan [36]	2016	Sample size calculation based on differences in hypothesis testing
17	Negida et al. [37]	2019	Sample size calculation based on sensitivity, specificity, and the area under the ROC curve

**Table 2:** Recommended sample size requirements for diagnostic research with various specifications of sensitivity, specificity, prevalence, and desired width that are based on 95% confidence interval.

Sensitivity	Specificity	Prevalence	Width	n^a^	n^b^	Prevalence	Width	n^a^	n^b^
0.95	0.95	0.05	0.05	6680	352				
0.90	0.90			11,860	625				
0.80	0.80			20,440	1076				
0.70	0.70			26,580	1399				
0.95	0.95		0.10	1880	99	0.5	0.10	188	188
0.90	0.90			3160	167			316	316
0.80	0.80			5280	278			528	528
0.70	0.70			6820	359			682	682
0.90	0.90		0.20	880	47		0.20	88	88
0.80	0.80			1400	74			140	140
0.70	0.70			1780	94			178	178
0.95	0.95	0.1	0.10	940	105	0.6	0.10	157	235
0.90	0.90			1580	176			264	395
0.80	0.80			2640	294			440	660
0.70	0.70			3410	379			569	853
0.90	0.90		0.20	440	49		0.20	74	110
0.80	0.80			700	78			117	175
0.70	0.70			890	99			149	223
0.95	0.95	0.2	0.10	470	118	0.7	0.10	135	314
0.90	0.90			790	198			226	527
0.80	0.80			1320	330			378	880
0.70	0.70			1705	427			488	1137
0.90	0.90		0.20	220	55		0.20	63	147
0.80	0.80			350	88			100	234
0.70	0.70			445	112			128	297
0.95	0.95	0.3	0.10	314	135	0.8	0.10	118	471
0.90	0.90			527	226			198	791
0.80	0.80			880	378			330	1321
0.70	0.70			1137	488			427	1706
0.90	0.90		0.20	147	63		0.20	55	221
0.80	0.80			234	100			88	351
0.70	0.70			297	128			112	446
0.95	0.95	0.4	0.10	235	157	0.9	0.10	105	941
0.90	0.90			395	264			176	1581
0.80	0.80			660	440			294	2641
0.70	0.70			853	569			379	3411
0.90	0.90		0.20	110	74		0.20	49	441
0.80	0.80			175	117			78	701
0.70	0.70			223	149			99	891

Note: n^a^ refers to sample size for sensitivity; n^b^ refers to sample size for specificity.

**Table 3:** Recommended sample size requirements involving research for a screening purpose that emphasizes a degree of sensitivity that is based on its 95% confidence interval.

Sensitivity	Specificity	Prevalence	Width	n^a^	n^b^	Prevalence	Width	n^a^	n^b^
0.95	0.50	0.05	0.05	6680	1657				
0.90	0.50			11,860	1657				
0.80	0.50			20,440	1657				
0.70	0.50			26,580	1657				
0.95	0.50		0.10	1880	424	0.5	0.10	188	804
0.90	0.50			3160	424			316	804
0.80	0.50			5280	424			528	804
0.70	0.50			6820	424			682	804
0.90	0.50		0.20	880	110		0.20	88	208
0.80	0.50			1400	110			140	208
0.70	0.50			1780	110			178	208
0.95	0.50	0.1	0.10	940	447	0.6	0.10	157	1005
0.90	0.50			1580	447			264	1005
0.80	0.50			2640	447			440	1005
0.70	0.50			3410	447			569	1005
0.90	0.50		0.20	440	116		0.20	74	260
0.80	0.50			700	116			117	260
0.70	0.50			890	116			149	260
0.95	0.50	0.2	0.10	470	503	0.7	0.10	135	1304
0.90	0.50			790	503			226	1304
0.80	0.50			1320	503			378	1304
0.70	0.50			1705	503			488	1304
0.90	0.50		0.20	220	130		0.20	63	347
0.80	0.50			350	130			100	347
0.70	0.50			445	130			128	347
0.95	0.50	0.3	0.10	314	575	0.8	0.10	118	2011
0.90	0.50			527	575			198	2011
0.80	0.50			880	575			330	2011
0.70	0.50			1137	575			427	2011
0.90	0.50		0.20	147	149		0.20	55	521
0.80	0.50			234	149			88	521
0.70	0.50			297	149			112	521
0.95	0.50	0.4	0.10	235	670	0.9	0.10	105	4021
0.90	0.50			395	670			176	4021
0.80	0.50			660	670			294	4021
0.70	0.50			853	670			379	4021
0.90	0.50		0.20	110	174		0.20	49	1041
0.80	0.50			175	174			78	1041
0.70	0.50			223	174			99	1041

Note: n^a^ refers to sample size for sensitivity; n^b^ refers to sample size for specificity.

**Table 4:** Recommended sample size requirements involving research for a screening purpose that emphasizes a degree of specificity that is based on its 95% confidence interval.

Sensitivity	Specificity	Prevalence	Width	n^a^	n^b^	Prevalence	Width	n^a^	n^b^
0.50	0.95	0.05	0.05	31,480	352				
0.50	0.90			31,480	625				
0.50	0.80			31,480	1076				
0.50	0.70			31,480	1399				
0.50	0.95		0.10	8040	99	0.5	0.10	804	188
0.50	0.90			8040	167			804	316
0.50	0.80			8040	278			804	528
0.50	0.70			8040	359			804	682
0.50	0.90		0.20	2080	47		0.20	208	88
0.50	0.80			2080	74			208	140
0.50	0.70			2080	94			208	178
0.50	0.95	0.1	0.10	4020	105	0.6	0.10	670	235
0.50	0.90			4020	176			670	395
0.50	0.80			4020	294			670	660
0.50	0.70			4020	379			670	853
0.50	0.90		0.20	1040	49		0.20	174	110
0.50	0.80			1040	78			174	175
0.50	0.70			1040	99			174	223
0.50	0.95	0.2	0.10	2010	118	0.7	0.10	575	314
0.50	0.90			2010	198			575	527
0.50	0.80			2010	330			575	880
0.50	0.70			2010	427			575	1137
0.50	0.90		0.20	520	55		0.20	149	147
0.50	0.80			520	88			149	234
0.50	0.70			520	112			149	297
0.50	0.95	0.3	0.10	1340	135	0.8	0.10	503	471
0.50	0.90			1340	226			503	791
0.50	0.80			1340	378			503	1321
0.50	0.70			1340	488			503	1706
0.50	0.90		0.20	347	63		0.20	130	221
0.50	0.80			347	100			130	351
0.50	0.70			347	128			130	446
0.50	0.95	0.4	0.10	1005	157	0.9	0.10	447	941
0.50	0.90			1005	264			447	1581
0.50	0.80			1005	440			447	2641
0.50	0.70			1005	569			447	3411
0.50	0.90		0.20	260	74		0.20	116	441
0.50	0.80			260	117			116	701
0.50	0.70			260	149			116	891

Note: n^a^ refers to sample size for sensitivity; n^b^ refers to sample size for specificity.

## Data Availability

Not applicable.
